# Ascorbic Acid and a Cytostatic Inhibitor of Glycolysis Synergistically Induce Apoptosis in Non-Small Cell Lung Cancer Cells

**DOI:** 10.1371/journal.pone.0067081

**Published:** 2013-06-11

**Authors:** Saleha B. Vuyyuri, Jacob Rinkinen, Erin Worden, Hyekyung Shim, Sukchan Lee, Keith R. Davis

**Affiliations:** 1 Owensboro Cancer Research Program, Owensboro, Kentucky, United States of America; 2 Department of Genetic Engineering, College of Biotechnology and Bioengineering, Sungkyunkwan University, Suwon, Republic of Korea; 3 Department of Pharmacology and Toxicology, James Graham Brown Cancer Center, University of Louisville, Louisville, Kentucky; H. Lee Moffitt Cancer Center & Research Institute, United States of America

## Abstract

Ascorbic acid (AA) exhibits significant anticancer activity at pharmacologic doses achievable by parenteral administration that have minimal effects on normal cells. Thus, AA has potential uses as a chemotherapeutic agent alone or in combination with other therapeutics that specifically target cancer-cell metabolism. We compared the effects of AA and combinations of AA with the glycolysis inhibitor 3-(3-pyridinyl)-1-(4-pyridinyl)-2-propen-1-one (3-PO) on the viability of three non-small cell lung cancer (NSCLC) cell lines to the effects on an immortalized lung epithelial cell line. AA concentrations of 0.5 to 5 mM caused a complete loss of viability in all NSCLC lines compared to a <10% loss of viability in the lung epithelial cell line. Combinations of AA and 3-PO synergistically enhanced cell death in all NSCLC cell lines at concentrations well below the IC_50_ concentrations for each compound alone. A synergistic interaction was not observed in combination treatments of lung epithelial cells and combination treatments that caused a complete loss of viability in NSCLC cells had modest effects on normal lung cell viability and reactive oxygen species (ROS) levels. Combination treatments induced dramatically higher ROS levels compared to treatment with AA and 3-PO alone in NSCLC cells and combination-induced cell death was inhibited by addition of catalase to the medium. Analyses of DNA fragmentation, poly (ADP-ribose) polymerase cleavage, annexin V-binding, and caspase activity demonstrated that AA-induced cell death is caused via the activation of apoptosis and that the combination treatments caused a synergistic induction of apoptosis. These results demonstrate the effectiveness of AA against NSCLC cells and that combinations of AA with 3-PO synergistically induce apoptosis via a ROS-dependent mechanism. These results support further evaluation of pharmacologic concentrations of AA as an adjuvant treatment for NSCLC and that combination of AA with glycolysis inhibitors may be a promising therapy for the treatment of NSCLC.

## Introduction

A unique characteristic of many tumor cells is increased glucose uptake and elevated aerobic glycolysis with a concomitant reduction in oxidative phosphorylation through the tricarboxylic acid (TCA) cycle. This remarkable metabolic reprogramming, known as the Warburg effect [1], represents a potential target for inhibiting the uncontrolled cell proliferation that is a hallmark of cancer. Initial explanations for the reliance of cancer cells on aerobic glycolysis suggested that cancer cells contained defective mitochondria and thus, enhanced glycolysis was required to generate ATP to drive cell proliferation. However, it is now known that most cancer cells have functional mitochondria, and that the metabolic changes associated with the Warburg effect are geared towards providing biosynthetic precursors for amino acids, nucleotides and lipids [1], [2]. In addition to driving increased glycolysis, the enhanced uptake of glucose characteristic of many cancer cells supports increased flux through the pentose phosphate shunt and the production of ribose-5-phosphate for nucleotide biosynthesis. Perhaps more importantly, increased flux through the pentose phosphate shunt can increase the amount of NADPH available to support metabolic activity and provide protection from oxidative stress. Additional NADPH and biosynthetic precursors are produced by the catabolism of glutamine [3]. Thus, the Warburg effect requires the highly coordinated control of glycolysis, the pentose phosphate shunt, glutaminolysis and the mitochondrial TCA cycle.

The unique dependence of cancer cells on glycolysis makes them vulnerable to therapeutic intervention with specific glycolysis inhibitors. Several glycolytic enzymes, including hexokinase II, lactate dehydrogenase A, and glucose-6-phosphate isomerase, are over expressed in tumor cells and serve as both facilitators and regulators of cancer progression [4], [5]. Various components of the glycolytic pathway have been targeted for therapy development, although very few have been evaluated in clinical trials. 2-Deoxy-D-glucose (2-DG), 3-bromopyruvate and lonidamine have been reported to be useful glycolytic inhibitors targeting hexokinase, the entry-point enzyme for glycolysis [5], [6]. 3-Bromopyruvate also inhibits glyceraldehyde-3-phosphate dehydrogenase (GAPDH) [6] and a recent study indicated that 3-bromopyruvate propyl ester was a more efficient inhibitor of GAPDH compared to hexokinase in colorectal carcinoma cells [7]. Another key glycolytic enzyme highly expressed in tumor cells is 6-phosphofructo-2-kinase/fructose-2,6-bisphosphatase isozyme 3 (PFKFB3), which generates fructose-2,6-bisphosphate (Fru-2,6-BP). Fru-2,6-BP relieves the repression of the key rate limiting enzyme 6-phosphofructo-1-kinase by ATP, thus allowing high rates of glycolysis in the presence of high ATP levels [8]. Small molecule inhibitors of PFKFB3 have been identified and shown to inhibit tumor cell growth [9], [10]. These novel inhibitors represent a new class of glycolysis inhibitors and further validate glycolysis inhibitors as potential cancer therapeutics, [4], [11].

Despite the dependence of cancer cells on glycolysis for ATP generation, inhibiting glycolysis using glycolytic inhibitors often does not prove to be effective in killing tumor cells as exemplified in a number of *in vivo* experiments [4], [5], [12]–[18]. This suggests that strategies aimed at inhibiting glycolysis may require multiple ATP depleting agents with different mechanisms of action [16] or that glycolysis inhibitors should be paired with other tumor-specific metabolism inhibitors. This approach has proven successful in a number of cases [12]–[15], [17], [18], suggesting that combination treatments using glycolytic inhibitors paired with other anticancer agents could be very powerful in the clinic.

Ascorbic acid (AA) has been shown to have cancer therapeutic potential; however, to date its therapeutic value remains controversial [19]–[23]. At lower concentrations, AA functions primarily as an antioxidant and can protect cells from oxidative stress whereas at higher concentrations AA acts as a pro-oxidant that imposes oxidative stress and induces cell death [20], [23]–[27]. It is likely that this concentration-dependent dual nature of AA is the basis for the inconsistent efficacy of AA in cancer therapy, since only pharmacologic concentrations of AA higher than those that can be obtained by oral delivery would likely exert anticancer effects [28]. AA has been shown to be selectively more toxic to cancer cells compared to corresponding normal cells [29]–[32]. A major component of this selective cytotoxicity is the ability of pharmacologic concentrations of AA to impose oxidative stress on cancer cells through the generation of ROS and hydrogen peroxide [33]–[35]. Since cancer cells generally have higher levels of reactive oxygen species, it appears that the additional oxidative stress imposed by AA cannot be ameliorated by cellular antioxidant responses and cell death is triggered [36]. Several studies have shown that combinations of AA with other anticancer agents often exhibit enhanced cytotoxicity [34], [37]–[40].

In this study, we determined that AA is selectively toxic to several non-small cell lung cancer (NSCLC) cell lines and that combination of AA and 3-(3-pyridinyl)-1-(4-pyridinyl)-2-propen-1-one (3-PO), a novel inhibitor of PFKFB3 with significant anticancer activity [9], synergistically induces apoptosis in NSCLC cells.

## Results

### AA and 3-PO Synergistically Inhibit Growth of NSCLC Cell Lines but not Bronchial Epithelial Cells

**Figure 1:**
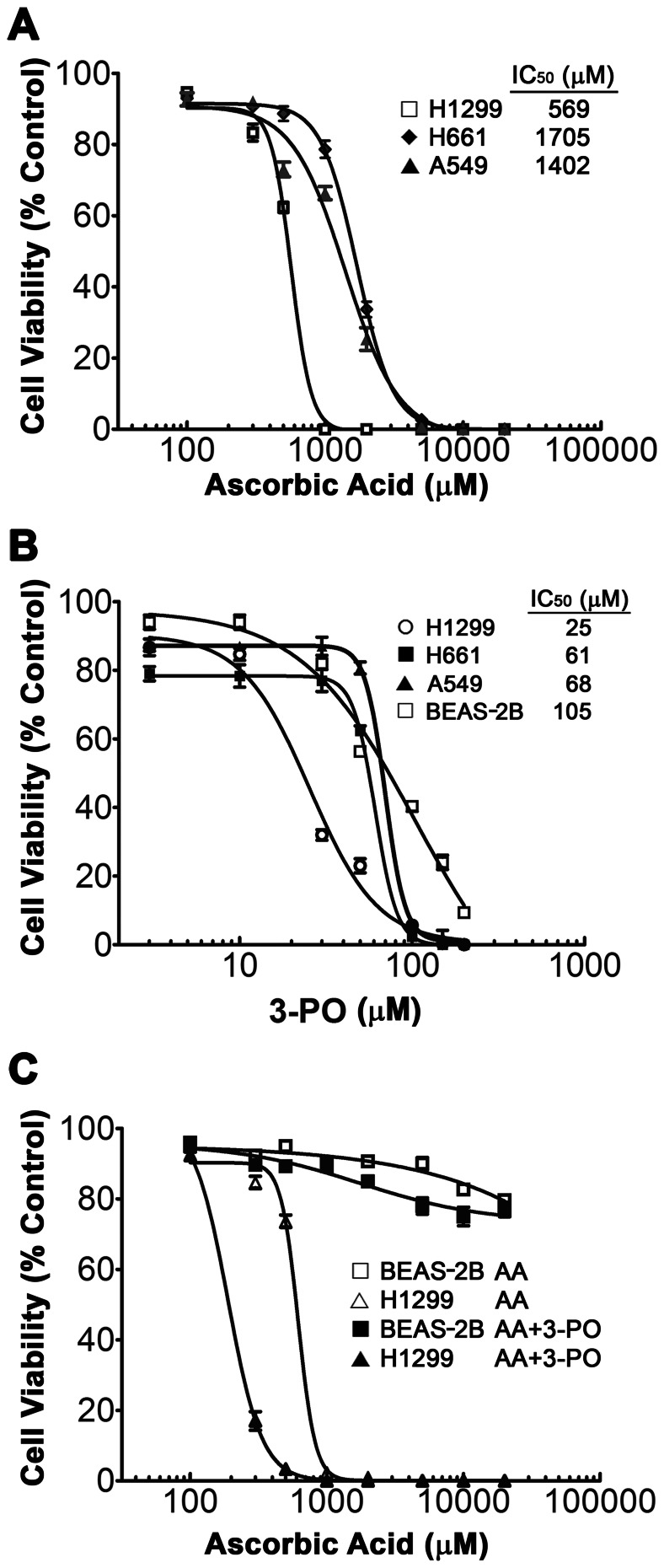
Effects of AA and 3-PO on the viability of NSCLC and normal lung epithelial cells. (A) Cell viability of NSCLC H1299, H661 and A549 cells as a function of AA concentration. (B) Cell viability of NSCLC H1299, H661 and A549 cells as a function 3-PO concentration. (C) Cell viability of NSCLC H1299 cells and BEAS-2B lung epithelial cells after treatment with AA alone and combinations of AA with 10 µM 3-PO. Cells were treated for 24 h and were then evaluated using the trypan blue exclusion viability assay and normalized to the appropriate vehicle-treated control. Data represent means ±SEM determined from three individual experiments. IC_50_ values shown were calculated using GraphPad Prism software.

Previous studies demonstrated that AA selectively decreases cell proliferation in some cancer cell lines without affecting normal cells [29]–[32]. We initiated studies to determine if this is indeed the case for NSCLC cells and to determine if combinations of AA with the glycolytic inhibitor 3-PO were more effective than AA alone. Initial studies were completed to determine the IC_50_ for AA and 3-PO in three NSCLC cell lines (H1299, H661, and A549) and a immortalized lung epithelial cell line (BEAS-2B) using a trypan blue exclusion cell viability assay. The 24 h IC_50_ concentrations for AA in the three NSCLC lines ranged from 0.57 to1.71 mM, with H1299 being the most sensitive (Fig. 1A). The BEAS-2B cells were much more tolerant to AA treatment, with an IC_50_ concentration >20 mM (Fig. 1C). These results demonstrated that NSCLC cells are considerably more sensitive to AA compared to BEAS-2B lung epithelial cells. The 24 h IC_50_ concentrations for 3-PO in the three NSCLC lines ranged from 25 to 67 µM, with H1299 again being the most sensitive (Fig. 1B). The IC_50_ concentration for BEAS-2B cells was 105 µM, demonstrating that the immortalized lung epithelial cells were 1.5 to 4.2-fold more resistant to 3-PO compared to the NSCLC cells.

We next investigated the effects of combinations of AA and 3-PO on the viability of the most sensitive cell line H1299 compared to BEAS-2B. For these experiments, cells were treated with 10 µM 3-PO, and concentrations of AA ranging from 0.1 to 20 mM. Combination treatments with AA and 3-PO did not have a marked effect on the viability of the BEAS-2B cells over the concentration ranges tested with the maximum decrease in viability of 18% ±1.3 observed in the 20 mM AA/10 µM 3-PO treatment (Fig. 1C). The Drewinko Index (DI) values for all of the AA and 3-PO combinations tested in BEAS-2B ranged from 1.0 to 1.1, indicating that the modest viability changes observed were due to the additive effects of 3-PO and AA. In contrast, combination treatments were significantly more effective in killing H1299 cells compared to AA alone (Fig. 1C). Treatment with 300 µM or 500 µM AA alone decreased cell viability by 15.3% and 26.3%, respectively, whereas in the presence of 10 µM 3-PO, 300 µM and 500 µM AA reduced viability by 84% and 96%, respectively. The combination treatment IC_50_ was 3-fold less than the IC_50_ of AA alone in H1299. The DI values for the combination treatments containing 300 µM and 500 µM AA were 4.9 and 18.7, respectively, and demonstrate a strong synergistic interaction between AA and 3-PO.

**Figure 2:**
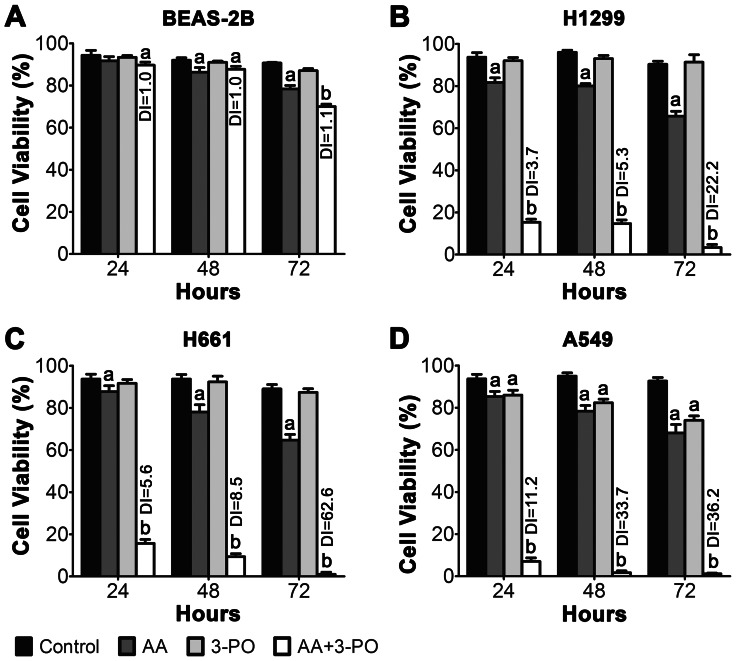
Comparisons of the effects of AA, 3-PO and combinations of AA and 3-PO on the viability of NSCLC and normal lung epithelial cells as a function of time after treatment. Lung epithelial cells BEAS-2B (A) and NSCLC cells H1299 (B), H661 (C), and A549 (D) were treated with 300 µM AA alone, 10 µM (BEAS-2B, H1299) or 30 µM (H661, A549) 3-PO alone, or a combination of AA and 3-PO. Control cells were treated with vehicle alone. At 24, 48 and 72 h post-treatment, cell viability was determined using the trypan blue exclusion assay. Data represent means ±SEM determined from three individual experiments. a; statistically significant difference (*P*<0.05) from vehicle treated control. b; statistically significant difference (*P*<0.05) from control and individual AA and 3-PO treatments. Drewinko Index (DI) values were calculated using the formula SF1 x SF2/SF1+2 where SF1, SF2 and SF1+2 represent the surviving fraction of cells treated with AA alone, 3-PO alone, and the combination of AA and 3-PO, respectively. DI values >1 indicates a synergistic effect, DI  = 1 indicates an additive effect; and DI values <1 indicates an antagonistic effect.

To determine if a similar synergistic interaction between AA and 3-PO occurred in other NSCLC lines, cell viability was compared in BEAS-2B, H1299, H661 and A549 cells treated with 300 µM AA, 10 µM (BEAS-2B, H1299) or 30 µM 3-PO (H661, A549), or a combination of AA with 3-PO over a 72 h period after treatment. As previously observed, these treatments had modest effects on the viability of BEAS-2B cells (Fig. 2A). The maximum loss of viability in BEAS-2B occurred at 72 h and was 30% with the combination treatment. The DI values for the combination treatment over the treatment period ranged from 1.0 to 1.1, confirming that there is not a synergistic interaction between AA and 3-PO in BEAS-2B cells. In contrast, combinations of AA and 3-PO synergistically killed all three NSCLC lines, with significant (*P*<0.05) synergistic (DI >1) effects clearly evident over the 72 h treatment period (Fig. 2B–D). The effects of the combination treatment on H1299 and H661 were similar, with a near complete loss of viability at 72 h. A549 cells were more sensitive to the combination treatment, with a near complete loss of viability at 48 h. The DI values for the combination treatment at 72 h ranged from 22.2 to 62.6 for the three NSCLC lines, indicating a strong synergistic interaction. These results demonstrate that a combination of AA and 3-PO synergistically induces cell death in multiple NSCLC lines and confirms that a similar interaction does not occur in the lung epithelial cell line BEAS-2B.

**Figure 3:**
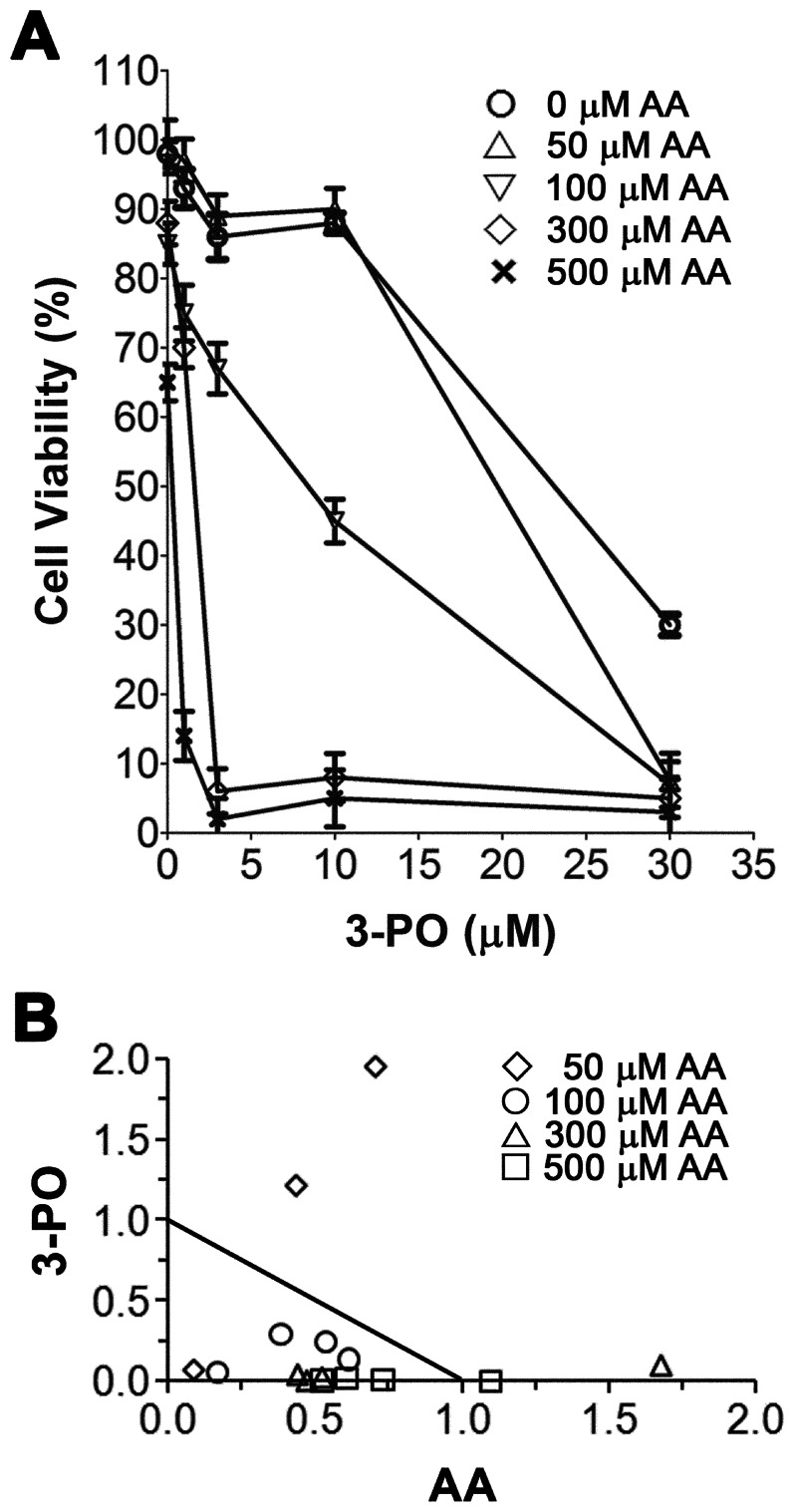
Effects of combinations of AA and 3-PO on the viability of NSCLC cells. (A) Cell viability as a function of 3-PO concentration in combination with AA concentrations ranging from 50 to 500 µM. (B) Normalized isobologram for each of the 3-PO and AA combinations tested. H1299 cells were treated with 3-PO (1, 3, 10, 30 µM) alone or in combination with different concentrations of AA (25, 50, 100, 300 or 500 µM). Cell viability was assessed 24 h after treatment using the trypan blue exclusion viability assay. Data represent means ±SEM determined from three individual experiments. The isobologram data were generated by CalcuSyn software using the non-constant ratio method and the data shown in (A). Symbols indicate values obtained in combination treatments containing the indicated concentration of AA.

Having established that AA and 3-PO cause a synergistic induction of cell death in NSCLC cell lines, we further investigated the interactions of a range of AA and 3-PO concentrations on H1299 cells (Fig. 3A). Concentrations of 1–10 µM 3-PO did not have a significant effect on the viability of H1299 cells, whereas 30 µM 3-PO caused a 60% loss in viability. Concentrations of AA from 50–500 µM showed a dose-dependent decrease in viability, with 500 µM causing a 40% loss in viability. A significant synergistic response was observed in H1299 cells over a broad range of AA and 3-PO combinations (Fig. 3B, Table 1). Combinations of 100, 300 and 500 µM AA with concentrations of 3-PO ≥ 3 µM caused the synergistic induction of cell death [Combination Index (CI) <1]. The combination treatment containing 50 µM with 30 µM 3-PO also caused a significant synergistic effect that resulted in >90% cell death (CI  = 0.16). Several combination treatments caused only additive effects or potentially antagonistic interactions (CI >1). However, these cases were only observed in combinations containing the lowest AA and 3-PO concentrations tested; these concentrations of 3-PO and AA alone caused less than a 5% loss of viability. These results demonstrate combinations of AA and 3-PO at concentrations more than 20-fold less than their respective IC_50_ resulted in the synergistic induction of cell death and a nearly complete loss of NSCLC H1299 cell viability.

**Table 1:** Combination index values for all combinations of 3-PO and AA.

	50 µM AA		100 µM AA		300 µM AA		500 µM AA	
3-PO (µM)	Fa^a^	CI^b^	Fa	CI	Fa	CI	Fa	CI
1	0.03	2.66	0.25	0.74	0.35	1.62	0.86	1.10
3	0.11	1.65	0.33	0.79	0.94	0.48	0.98	0.53
10	0.10	5.03	0.55	0.69	0.92	0.55	0.97	0.62
30	0.92	0.16	0.94	0.21	0.97	0.39	0.99	0.42

a Fraction affected;

b Combination index as determined by the median effect method [42] using CalcuSyn software: CI <1 indicates synergistic, CI  = 1 indicates additive, and CI >1 indicates antagonistic compound interactions.

### Contribution of ROS and H_2_O_2_ to the Mechanism of Cell Death Induction by Combinations of AA with 3-PO

**Figure 4:**
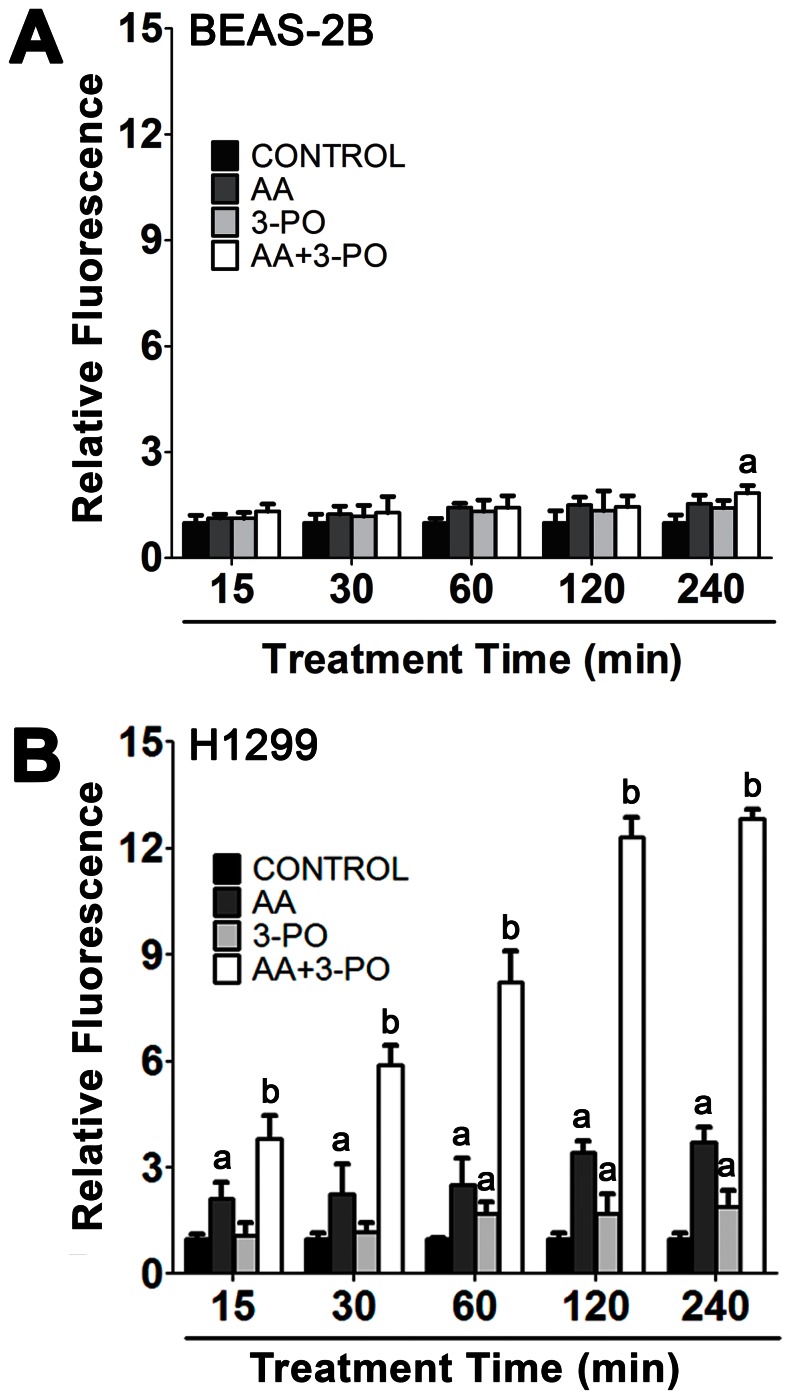
Effects of combination of AA and 3-PO on ROS accumulation in lung epithelial and NSCLC cells. BEAS-2B (A) and H1299 (B) cells were treated with either AA (300 µM), 3-PO (10 µM) or a combination of AA and 3-PO for the indicated times. Cells were stained with 5 µM CM-H_2_DCFDA at 37°C in the dark for 30 min to detect intracellular ROS. Data represent means ±SEM from two independent experiments where 100–125 cells per sample were analyzed for fluorescence intensity. a; statistically significant difference (P<0.05) from vehicle treated control. b; statistically significant difference (P<0.05) from control and individual AA and 3-PO treatments.

Previous studies have established that ROS and H_2_O_2_ produced through the oxidation of AA is a crucial mediator of AA-induced cytotoxicity [29], [31], [33], [41], [42]. We assessed the potential importance of ROS production in the sensitivity of NSCLC cells to combinations of AA and 3-PO by treating BEAS-2B and H1299 cells with a combination of 10 µM 3-PO with 300 µM AA and measuring cellular fluorescence of the ROS reporter 5-(and-6)-chloromethyl-2′,7′-dichlorodihydrofluorescein diacetate, acetyl ester (CM-H_2_DCFDA). ROS levels were not significantly affected in BEAS-2B cells by either AA or 3-PO alone over a 4 h treatment period, or in combination treatments of 2 h or less. The only statistically significant (*P*<0.05) effect observed in BEAS-2B cells occurred with the combination treatment at 4 h where a 51% increase in ROS was observed (Fig. 4A and Fig. S1). A significant (*P*<0.05) increase in ROS levels was observed as early as 15 min in H1299 cells treated with either AA or the combination of 3-PO and AA, with the combination treatment level being about 2-fold higher than AA alone (Fig. 4B and Fig. S1). ROS levels in AA-treated cells continued to rise modestly and were 4-fold higher than the vehicle-treated control at 4 h. 3-PO also induced low levels of ROS production between 1 and 4 h of treatment that were 2-fold higher than the control. In contrast, the combination treatment induced much higher ROS levels that reached a maximum at 2–4 h; these levels were 13-fold higher than the control and significantly higher than the sum effects of the individual AA and 3-PO treatments. The ROS levels in H1299 cells at 4 h were 7-fold higher than combination-treated BEAS-2B cells. These results demonstrate that combinations of AA and 3-PO selectively induce high levels of ROS in H1299 compared to BEAS-2B and suggest that ROS production is a component of the synergistic induction of combination-induced cell death observed in NSCLC cells.

**Figure 5:**
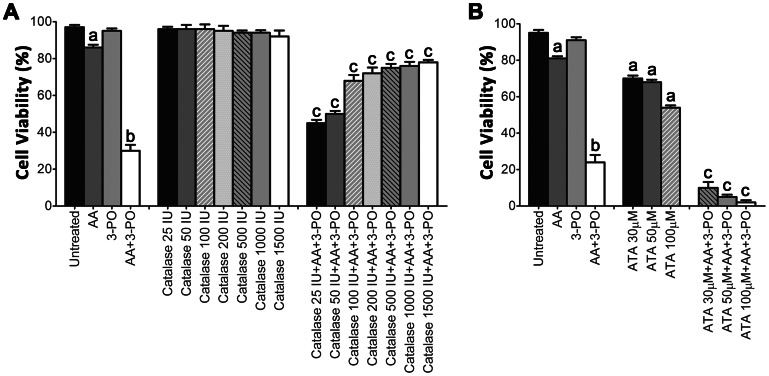
Identification of H_2_O_2_ as a mediator of the synergistic induction of cell death in NSCLC cells by combination of AA and 3-PO. (A) Reduction of combination-induced cell death by addition of catalase to the culture medium. H1299 cells were treated with either AA alone (300 µM), 3-PO alone (10 µM), a combination of AA and 3-PO, catalase alone (25, 50, 100, 200, 500, 1000, 1500 IU), or combinations of AA, 3-PO and catalase. (B) Enhancement of combination-induced cell death by addition of the catalase inhibitor aminotriazole (ATA) to the culture medium. H1299 cells were treated with either AA alone (300 µM), 3-PO alone (10 µM), a combination of AA and 3-PO, ATA alone (30, 50, 100 µM), or combinations of AA, 3-PO and ATA. Cells were assessed 24 h after treatment using the trypan blue exclusion viability assay. Data represent means ±SEM determined from three individual experiments. a; statistically significant difference (*P*<0.05) from vehicle treated control. b; statistically significant difference (*P*<0.05) from control and individual AA and 3-PO treatments. c; statistically significant difference (*P*<0.05) from AA and 3-PO combination treatment.

To determine the degree to which H_2_O_2_ mediated the effect of AA in sensitizing NSCLCs to 3-PO, H1299 cells were treated with a combination of 10 µM 3-PO with 300 µM AA in either the presence or absence of catalase in the culture medium. A significant (*P*<0.05) dose-dependent reversal of the cytotoxic effect of the AA and 3-PO combination was observed over a concentration range of 25 to 1500 units of catalase (Fig. 5A). The rescue by catalase was not complete; the inhibition of cell death plateaued at ∼80% viability. The role of H_2_O_2_ in mediating the synergistic activation of cell death by a combination of AA and 3-PO was further tested by pre-incubating cells with aminotriazole, a well-known catalase inhibitor, prior to treatment with AA and 3-PO. A significant (*P*<0.05) dose-dependent enhancement of cell death compared to aminotriazole alone was observed over an aminotriazole concentration range of 30 to 100 µM (Fig. 5B). This demonstrates that inhibition of endogenous catalase activity enhances the synergistic induction of cell death by a combination of AA and 3-PO. Taken together, these studies demonstrate the mechanistic importance of H_2_O_2_ in the synergistic induction of cell death in NSCLC cells by co-treatment with AA and 3-PO.

### Combination of AA and 3-PO Induces Apoptosis in NSCLC Cells

**Figure 6:**
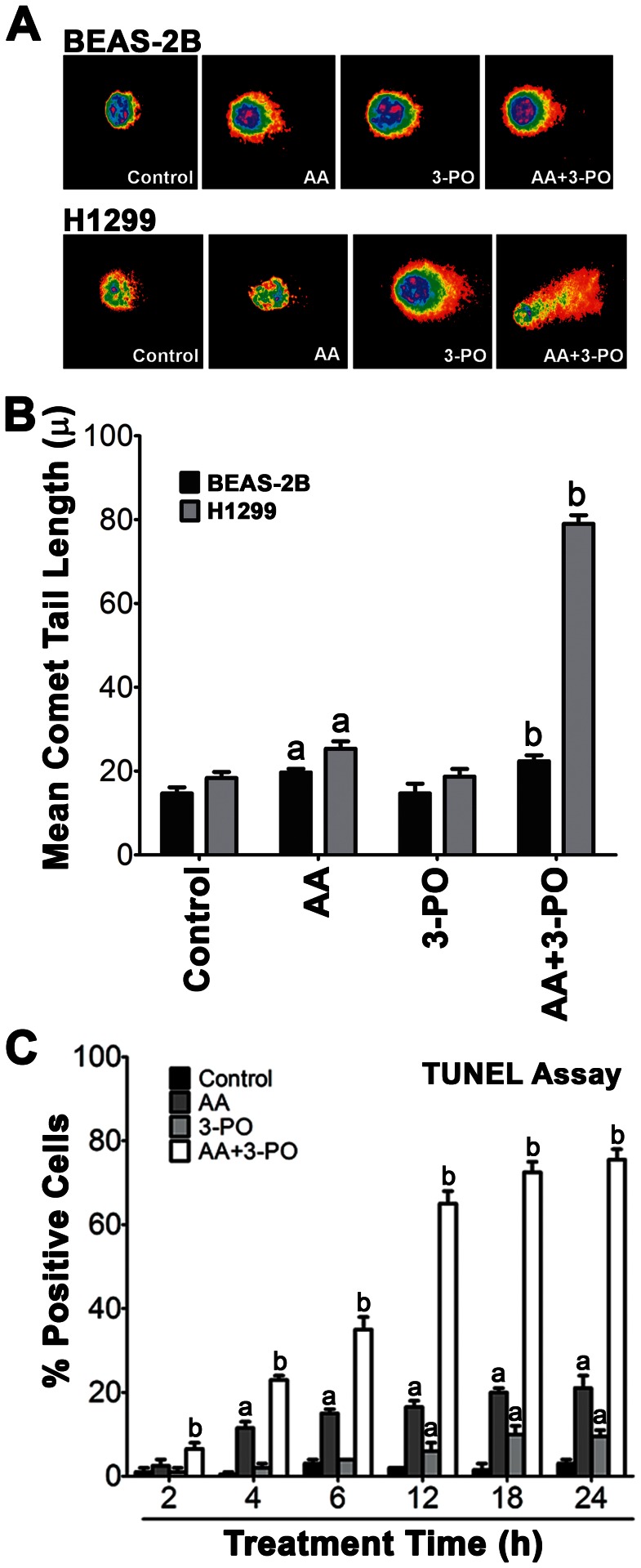
Enhanced induction of DNA fragmentation in NSCLC cells by combination of AA and 3-PO. (A) Comet assay images of BEAS-2B lung epithelial cells and H1299 cells treated with either AA (300 µM), 3-PO (10 µM), or a combination of AA and 3-PO. Cells were processed 24 h after treatment and vehicle-treated cells were included as a control. (B) Quantitation of DNA fragmentation detected by the comet assay in BEAS-2B lung epithelial cells and H1299 cells treated with either AA, 3-PO, or the combination of AA and 3-PO. Cells were processed 24 h after treatment. Mean comet tail length data represent means ±SEM determined from three independent experiments were the tail length of 150 cells per treatment was measured in each experiment. (C) Quantitation of DNA fragmentation by TUNEL assays. H1299 cells were treated with either AA (300 µM), 3-PO (10 µM), or a combination of AA and 3-PO for the indicated times. TUNEL positive cells were identified using the DeadEndTM Fluorometric TUNEL System. Data represent means ±SEM from two independent experiments where a minimum of 100 cells per sample were visualized in each experiment. a; statistically significant difference (P<0.05) from vehicle treated control. b; statistically significant difference (P<0.05) from vehicle treated control and individual AA and 3-PO treatments.

Microscopic examination of NSCLC cells treated with combinations of AA and 3-PO revealed that treated cells exhibited the classic morphological changes associated with apoptosis including cell shrinkage, blebbing and the formation of apoptotic bodies. To further investigate whether the synergistic induction of cell death in NSCLC cells by combination of 3-PO and AA was indeed due to apoptosis, we assessed DNA fragmentation, a hallmark of cells undergoing apoptosis. DNA fragmentation was first measured using the comet assay in NSCLC H1299 cells and BEAS-2B lung epithelial cells treated with AA or 3-PO alone and in combination (Fig. 6A and B). Treatment with 10 µM 3-PO alone did not cause a significant increase in DNA fragmentation in either cell line whereas 300 µM AA caused a modest, but significant (*P*<0.05) increase in both BEAS-2B and H1299 cells at 24 h after treatment. The combination of 3-PO and AA did not cause significantly more DNA fragmentation in the BEAS-2B cells than that observed in the cells treated with AA alone. In contrast, the amount of DNA fragmentation observed in the H1299 cells was significantly (*P*<0.05) higher in the combination treatment (75.02±2.29) compared to the individual treatments and to the control (AA 25.63±2.38, 3-PO 19.63±2.24, control 17.96±1.66) and was significantly higher than the sum effects of the individual AA and 3-PO treatments.

Additional studies of DNA fragmentation in H1299 cells were performed using a terminal deoxynucleotidyl transferase dUTP nick end labeling (TUNEL) assay to assess the kinetics of DNA fragmentation during the first 24 h of treatment (Fig. 6C). Modest but significant (*P*<0.05) increases in DNA fragmentation were observed in cells treated with either AA or 3-PO alone starting at 4 and 12 h, respectively. After 24 h of treatment, the amount of DNA fragmentation observed in AA-treated and 3-PO-treated cells was 6-fold and 2.5-fold higher, respectively, compared to the vehicle-treated control. Combination of AA and 3-PO induced a significantly higher (*P*<0.05) level of DNA fragmentation compared to the control and individual treatments as early as 2 h after treatment. DNA fragmentation in combination-treated cells began to plateau at 12 h after treatment and was 20-fold higher than the control at 24 h after treatment. The level of DNA fragmentation observed in the combination-treated cells at all the time points tested was also significantly higher than the sum effects of the individual AA and 3-PO treatments. These results are consistent with the comet assay studies and suggest that the induction of apoptosis is a component of combination-induced cell death in H1299 cells.

**Figure 7:**
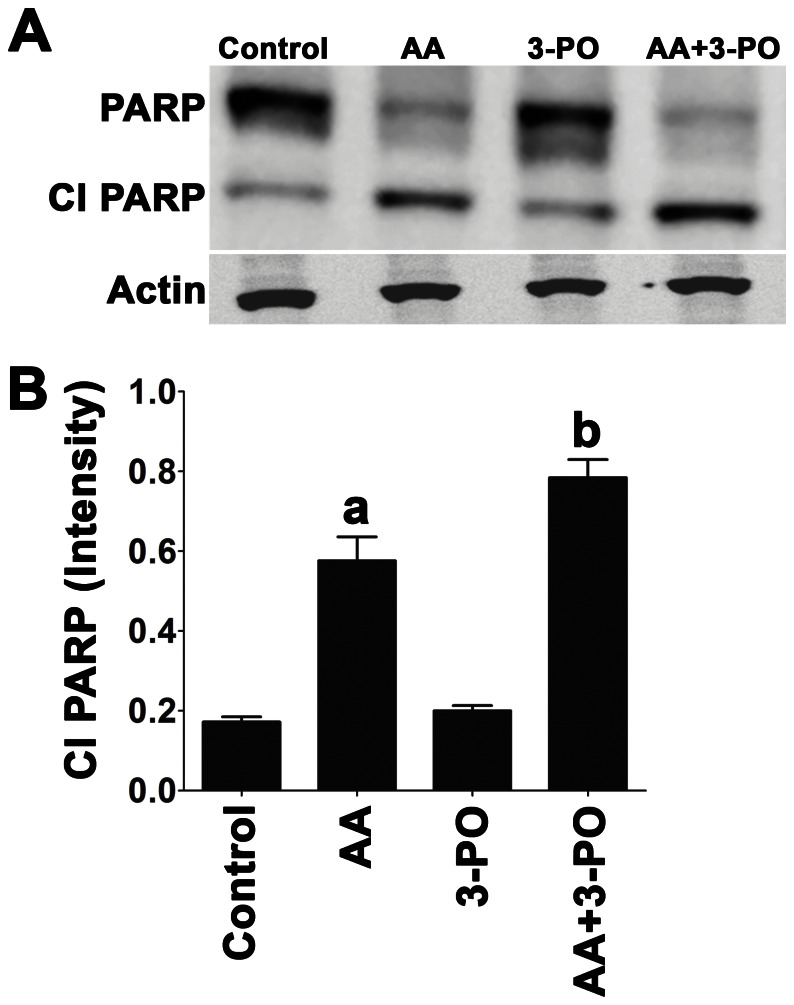
Induction of PARP cleavage in H1299 cells by combination of AA and 3-PO. (A) H1299 cells were treated with either AA (300 µM), 3-PO (10 µM), or the combination of AA and 3-PO. Cells were harvested 24 h after treatment and protein extracts subjected to immunoblot analysis using a PARP-specific antibody. Duplicate immunoblots probed with an actin-specific antibody served as normalization controls. (B) Quantitation of the levels of cleaved PARP (Cl PARP) was done using image analysis of the immunoblots using Carestream Molecular Imaging Software version 5.0. The intensity measurements shown represent means ±SEM determined from three independent experiments. a; statistically significant difference (*P*<0.05) from vehicle treated control. b; statistically significant difference (*P*<0.05) from control and individual AA and 3-PO treatments.

To further evaluate if the cell death induced by combination of AA and 3-PO was related to the induction of apoptosis, we examined the extent of PARP cleavage. PARP is a 116 kDa stress-response protein involved in the repair of damaged DNA and regulates chromatin structure by poly (ADP-ribosylation) of nuclear proteins. Proteolytic cleavage of PARP into 89 kDa and 24 kDa fragments by caspases is an indicator of apoptosis [43]. The levels of cleaved PARP were measured by immunoblotting cell lysates 24 h after treatment with 300 µM AA, 10 µM 3-PO or a combination AA and 3-PO for 24 h (Fig. 7A and B). Treatment with 3-PO alone did not cause a significant increase in cleaved PARP (89 kDa fragment). An increase in the accumulation of cleaved PARP was observed in cells treated with AA alone or the combination of AA and 3-PO that was significantly (*P*<0.05) higher than the vehicle control. The extent of PARP cleavage in cells treated with the combination of AA and 3-PO was significantly (*P*<0.05) higher than in cells treated with AA alone.

**Figure 8:**
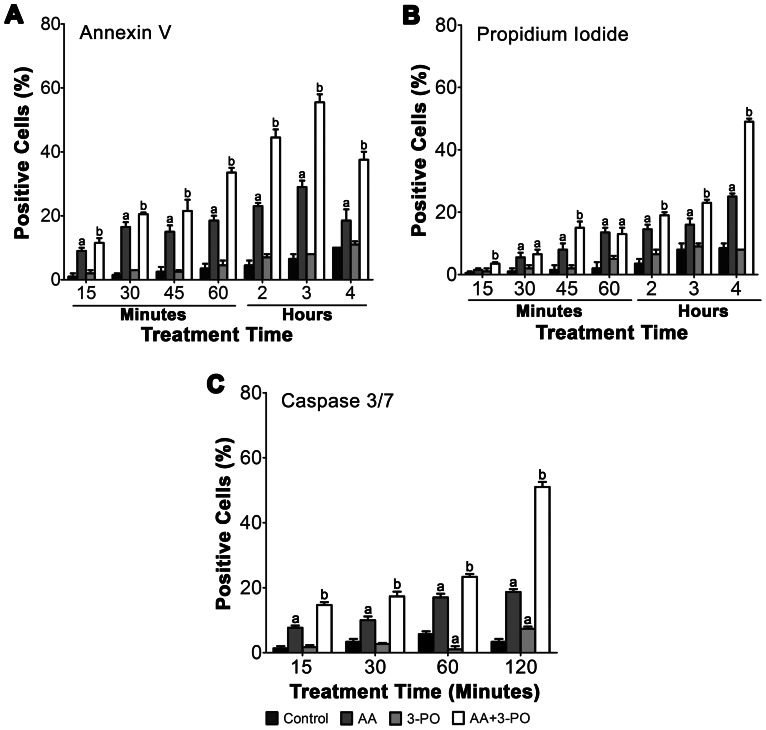
Enhanced induction of apoptosis markers in NSCLC cells by combination of AA and 3-PO. (A) Analysis of early apoptotic cells and (B) late apoptotic cells by annexin V binding and propidium idodide staining. H1299 cells were treated with either AA (300 µM), 3-PO (10 µM) or a combination of AA and 3-PO for the indicated times. Cells were then stained with annexin V and propidium iodide and visualized by fluorescent microscopy. A minimum of 200 cells were scored for each sample and the data represent means ±SEM from three independent experiments. (C) Analysis of caspase 3/7 activity. H1299 cells were treated with either AA (300 µM), 3-PO (10 µM) or a combination of AA and 3-PO for the indicated times. Caspase 3/7-positive cells were identified using a fluorogenic caspase substrate specific for activated caspase 3 and/or 7 (CellEvent™ Caspase-3/7 Green). Data represent means ±SEM from three independent experiments where 150–200 cells per sample were visualized in each experiment. a; statistically significant difference (*P*<0.05) from vehicle treated control. b; statistically significant difference (*P*<0.05) from control and individual AA and 3-PO treatments.

One of the early events of apoptosis involves the translocation of phosphatidylserine to the surface of the cell which can be detected using assays based on annexin V binding. Thus, to further assess whether cell death induced in H1299 cells by combination treatments of AA and 3-PO is due to apoptosis, treated cells were analyzed for annexin V binding and propidium iodide staining to measure early apoptotic (annexin V+/propidium iodide –) and late apoptotic (annexin V +/propidium idodide +) cell populations (Fig. 8A and B). Treatment with 10 µM 3-PO alone did not have a significant effect on the number of early or late apoptotic cells compared to the control. Treatment with 300 µM AA induced a significant (*P*<0.05) gradual increase in both early and late apoptotic cells. Early apoptotic cells were detected as soon as 15 min after treatment and reached a maximum of 29% at 3 h post-treatment. The percentage of early apoptotic cells declined at 4 h. A similar gradual accumulation of late apoptotic cells occurred in AA-treated cells over the 4 h treatment period, with the first detectable significant difference from the control observed at 30 min and reached 18% at 4 h. The combination treatment also exhibited a gradual increase in early apoptotic cells from 15 min to 3 h, however, the percentage of early apoptotic cells was significantly higher than that observed with AA alone and reached a maximum of 56% at 3 h post-treatment. Similar to the AA alone treatment, the number of early apoptotic cells decreased with a concomitant increase in late apoptotic cells at 4 h. The percentage of late apoptotic cells at 4 h post-treatment was 50% and was significantly (*P*<0.05) higher than the 26% observed in cells treated with AA alone.

To confirm that the combination of AA and 3-PO induces cell death via an apoptotic mechanism, we measured changes in caspase activity in H1299 cells using a fluorometric assay that detects active caspases 3 and 7 (Fig. 6C). Cells treated with 10 µM 3-PO showed little or no change in caspase activity whereas 300 µM AA induced significantly (*P*<0.05) higher caspase activity that was 3- to 5-fold higher than the control over the entire 2 h treatment period. The combination treatment also showed a significant induction of caspase activity over the entire treatment period, ranging from 4- to 16-fold higher than the control. The induction of caspase activity by the combination treatment was significantly (*P*<0.05) higher than observed with AA alone at all the treatment times tested. The induction observed at 2 h by the combined treatment (51% ±2.63) was higher than the sum effects of AA (19% ±3.0) and 3-PO alone (8% ±1.1). These results in conjunction with the observed induction of DNA fragmentation, PARP cleavage, and annexin V binding demonstrate that the synergistic induction of cell death by combination of AA and 3-PO is mediated at least in part by apoptosis.

## Discussion

The use of pharmacologic doses of AA as a cancer therapy has been controversial due to the lack of efficacy in earlier clinical trials [44], [45] and concern that the transported form of AA, dehydroascorbic acid, may protect cancer cells in the context of treatment with other chemotherapy drugs [24]. More recently, it has become clear that parenteral administration of AA results in much higher therapeutic doses than can be achieved using the oral administration methods utilized in these earlier clinical trials [23], [34], [46] and that these doses are effective alone or in combination with other anticancer agents in inducing cell death in several *in vitro* and *in vivo* cancer models [13], [29], [47]–[51]. Moreover, it has been shown that pharmacologic doses of AA exert effects through an extracellular autooxidation mechanism involving the ascorbyl radical and the subsequent generation of H_2_O_2_ [33]–[35] rather than conversion to dehydroascorbic acid. Taken together, an increasing body of evidence suggests that high-dose AA may serve as an important adjuvant treatment for treating a variety of cancers. Our data using several NSCLC cell lines clearly support this hypothesis and suggest that high-dose AA may be useful in the treatment of lung cancer.

Pharmacologic doses of AA at low mM concentrations were found to be selectively toxic to three different NSCLC cell lines compared to an immortalized lung epithelial cell line (Fig. 1). The IC_50_ values for these lines were at least an order of magnitude lower than the IC_50_ observed for the lung epithelial line. This is consistent with previous studies that showed normal human cell lines were much more tolerant to AA treatment compared to most cancer cell lines [48]. The IC_50_ values for a 24 h treatment of the NSCLC cell lines were very similar or lower than the IC_50_ values previously determined for human cancer cell lines representing a significant number of cancer types including leukemia, pancreatic, ovarian, breast, cervical, uterine, bladder, prostate, mesothelioma, liver, colon, gastric, renal, melanoma, glioblastoma, neuroblastoma, and lymphoma [31], [35], [38], [41], [47]–[50], [52]–[55]. Previous studies also reported that AA inhibited the growth of two NSCLC lines. The 48 h IC_50_ for A549 was ∼2 mM [47] whereas the IC_50_ for H1299 was >20 mM [48]. The previous results with A549 are very similar to our findings; however, our results indicate that H1299 is much more sensitive to AA treatment than found by Chen et al. [48]. One potential explanation is that the treatment protocols were different. Chen et al. [48] treated the cells with AA for two hours, then washed the cells and maintained them in AA-free media for the duration of the experiment. We treated the cells with AA and did not change the medium; consequently the cells may have been exposed to AA for a longer period. However, previous studies have shown that AA has a half-life of ∼2 h in culture medium under standard culture conditions, and likely even shorter in the presence of cells [56], [57], thus the actual exposure time of cells to AA in our experiments likely does not represent a major difference with these previous studies. It is also possible that differences in media conditions are responsible for the different sensitivities observed [35]. Our results demonstrate that NSCLC cells are as sensitive to AA as many other cancer types and highlight the common observation that AA concentrations between 0.5 and 10 mM are generally effective against a broad range of cancer types *in vitro*. This is a dose range that is readily achievable and well toleated in cancer patients [34], . In several cases, this *in vitro* sensitivity has been confirmed with human and murine cancer cell lines *in vivo* using mouse models [34], [48], [51], [59].

A key novel finding in our studies is that combination of AA with the cytostatic glycolysis inhibitor, 3-PO, synergistically induced cell death in all three NSCLC cell lines tested. In H1299 cells, the addition of 10 µM 3-PO with AA reduced the 24 h IC_50_ for AA by 3-fold (Fig. 1C). Similar strong synergistic interactions were also observed in the H661 and A549 cell lines. In contrast, the combination treatment caused only a 30% loss of viability in the BEAS-2B lung epithelial cell line and the DI values for the combination treatments indicate an additive effect (Fig. 2A). These results suggest that the synergistic induction of cell death induced by combinations of AA and 3-PO is selective for the NSCLC cells relative to lung epithelial cells and provide a preliminary indication that a similar combination treatment will not have toxicity issues *in vivo*.

A more detailed analysis of the effects of the combination of AA and 3-PO over concentration ranges of 50 to 500 µM AA and 1 to 30 µM 3-PO revealed that the synergistic induction of cell death could be obtained by increasing the concentration of either compound relative to the other over these concentration ranges (Fig. 3A–B, Table 1). Most combinations had CI values consistent with synergism (CI  = 0.3–0.7) with several combinations showing strong synergism (CI  = 0.1–0.3) [60]. Several combination treatments had CI values consistent with additive or antagonistic interaction; however, all of these combinations included either 1 µM 3-PO, 50 µM AA, or both. These concentrations of 3-PO and AA alone cause less than 5% cell death and were not significantly different from vehicle-treated controls. Thus, these CI values are unlikely to accurately reflect the ability of 3-PO and AA to interact. In addition, it is possible that at 50 µM, AA exerts its well know antioxidant effect that would be expected to promote cell growth. Taken together, these results confirm and extend previous studies that demonstrate the ability of AA to enhance the activity of other anticancer compounds. AA has been reported to enhance the anticancer activity of doxorubicin, cisplatin and paclitaxel in human breast cancer cells with a clear synergism observed with a combination of AA and doxorubicin [38]. More recently it has been shown the AA synergistically enhances cell death induced by gemcitabine in pancreatic cancer cells [51] and that a combination of AA and arginine synergistically induce apoptosis in a hepatoma cell line [40]. Our studies represent the first example of AA synergistically increasing the anticancer activity of a glycolysis inhibitor.

Initial mechanistic studies of the synergistic activation of cell death by combinations of AA and 3-PO revealed that production of ROS and H_2_O_2_ is likely required (Figs. 4A, 5 and Fig. S1). These results are consistent with previous studies demonstrating that pharmacologic concentrations of AA exert effects through the production of ROS and H_2_O_2_ and the corresponding cellular toxicity imposed by this oxidative stress [29], [31], [33], [35], [61]. The production of H_2_O_2_ initiated by treatment with AA has been proposed to occur via an extracellular mechanism both *in vitro* and *in vivo* [29], [33], [62]. In combination-treated H1299 cells, ROS production was rapidly induced with significantly higher levels than control or the single treatments observed as early as 15 min after treatment. Both AA and 3-PO individually induced some ROS production; however, the maximum levels observed were 4- to 7-fold less than that observed in the combination treatment. ROS levels in lung epithelial cells were not increased by treatment with either AA or 3-PO (Fig. 4B and Fig. S1). The combination treatment did induce a modest increase in ROS levels in lung epithelial cells after 4 h; however, this ROS induction was 7-fold less than that induced in H1299 cells. Based on previously published studies [29], [31], [33], [41], [42], it is likely that the induction of ROS in combination-treated cells resulted in the significant H_2_O_2_ accumulation. Our observations that addition of the impermeant H_2_O_2_ scavenger catalase to the medium reduced combination-induced cell death in NSCLC cells by as much as 70% and that combination-induced cell death was enhanced by inhibition of endogenous catalase by aminotriazole are consistent with this hypothesis. The inability to completely prevent combination-induced cell death by catalase is likely due to the fact that H_2_O_2_ easily crosses the plasma membrane [63], [64] and a portion of the H_2_O_2_ escapes degradation. It is also possible that other cytotoxic mechanisms unrelated to H_2_O_2_ are also induced by the combination treatment.

Further studies of the mechanism of cell death induced by the combination of AA and 3-PO clearly demonstrated that the induction of apoptosis was a key component of this response. Significantly higher levels of DNA fragmentation were observed in combination-treated cells compared to control cells and cells treated with either AA or 3-PO individually (Fig. 6). Significant DNA fragmentation was observed in combination-treated cells as early as 2 h after treatment and increased rapidly until 12 h, where upon DNA fragmentation began to plateau. The level of DNA fragmentation observed in the combination-treated cells was significantly higher than the predicted sum effects of the individual treatments and paralleled the synergistic induction of cell death. PARP cleavage, another marker for apoptosis indicative of caspase activation, was also significantly higher in combination-treated cells compared to control cells and cells treated with either AA or 3-PO individually (Fig. 7). Analysis of annexin V and caspase 3/7 levels demonstrated that these apoptosis markers were significantly induced by both AA alone and in the combination treatment as early as 15 min after treatment (Fig. 8A and 8C). However, combination-treated cells exhibited significantly more annexin V and caspase 3/7 activity compared to AA alone at all exposure times tested. At the later exposure times, the combination treatment induced annexin V binding and caspase 3/7 activity more than the predicted sum effects of the individual treatments. Taken together, these results demonstrate that the combination of AA with 3-PO preferentially induce cell death in NSCLC cells, at least in part, by the induction of apoptosis. This is consistent with previous studies that demonstrated AA alone induces apoptosis in other cancer cell types [41], [50], [52], [54] and that combinations of AA with arginine-induced hepatoma cell death via apoptosis [40].

Additional experiments are required to determine the precise mechanism and signaling pathways required for the synergistic induction of apoptosis by the combination of AA with 3-PO. It is important to consider that AA alone can induce apoptosis whereas 3-PO is cytostatic. We did observe a modest, but significant induction of cell death and apoptosis markers by AA alone at concentrations that were effectively synergistic when combined with 3-PO. This suggests that the addition of 3-PO to AA enhanced the activation of AA-induced apoptosis. 3-PO was previously shown to reduce NAD levels [9] and a modest reduction of NAD may have contributed to the synergistic induction of cell death observed in our studies. One unexplained aspect of our results is that it would be expected that inhibition of glycolysis would result in increased flux of glucose-6-phosphate into the pentose phosphate pathway and a concomitant increase in NADPH [1]. This would lead to increased glutathione and additional protection from oxidative stress. It appears that any increase in NADPH and glutathione that may have occurred via this mechanism was not sufficient to protect against the large increase in ROS induced by the combination treatment. Future metabolomic studies should provide a more detailed mechanistic description of the specific pathways affected by the combination of AA with 3-PO and provide new insight into the mechanisms causing the synergistic activation of cell death.

Our data suggest that p53 is not essential for the ability of AA or the combination treatment to induce cell death in NSCLC cells. The synergistic activation of cell death was observed in all three lines irrespective of p53 status, albeit, the lack of p53 sensitizes cells to both the single and combination treatments. The p53- cell line H1299 had an IC_50_ for AA alone that was 2.5- to 3-fold less than the IC_50_ values for the two p53+ lines A549 and H661. This is consistent with previous studies showing that p53- cells are often sensitized to drugs that induce metabolic stress [65]. Our results suggest that the apoptosis observed in cells exposed to AA or a combination of AA and 3-PO is regulated at least in part by a p53-independent apoptosis pathway. Other studies indicate that AA can inhibit cancer cell growth by inducing cell cycle arrest, autophagy and necrosis [29], [54], [62], [66], [67]. The ability of AA to affect cell cycle progression has been documented in several cell types [54], [66], [67]. The ability of AA to cause cell cycle arrest is likely mediated by cAMP signaling pathways that regulate key components of the cell cycle machinery [68]. Chen et al. [29] reported that in human lymphoma cells, lower concentrations of AA induced apoptosis whereas higher concentrations induced necrosis. This is similar to the aforementioned studies on cell cycle arrest where lower concentrations of AA induced S-phase arrest in proliferative normal and cancer cells whereas higher concentrations induced necrotic cell death [66]. In our studies, a combination of 10 µM 3-PO and 300 µM AA rapidly induced many of the markers of apoptosis in H1299 cells, suggesting that a major driver of the synergistic activation of cell death observed was due to apoptosis. It is possible that necrosis was also involved, particularly at later treatment times when ATP levels may be depleted. A number of studies have shown that various stresses induce an initial apoptotic response that transitions to necrosis when the cell no longer has the energy reserves required to fuel its orderly destruction (reviewed in [69], [70]).

In summary, we have demonstrated that NSCLC cells are significantly more sensitive to treatment with AA compared to normal lung epithelial cells and that combination of sublethal concentrations of AA with concentrations of the cytostatic glycolysis inhibitor 3-PO that cause only a modest inhibition of cell proliferation synergistically induce apoptosis in NSCLC. These results further support the growing body of evidence that pharmacologic doses of AA may be useful in treating a number of different cancers, including NSCLC. Moreover, our studies indicate that combination treatments using AA and glycolysis inhibitors may be a particularly effective cancer therapy for some difficult to treat malignancies.

## Materials and Methods

### Reagents

Media, media supplements, trypsin-EDTA, phosphate-buffered saline (PBS) and the Caspase 3–7 Green Detection Reagent were obtained from Life Technologies/Invitrogen (Grand Island, NY). BD Falcon cultureware was purchased from BD Biosciences (Franklin Lakes, NJ). Ascorbic acid, trypan blue, aminotriazole, catalase, antibiotics, buffer components, standard agarose and low-melting point agarose were purchased from Sigma-Aldrich (St. Louis, MO). Apoalert Annexin V kit was purchased from Clontech Laboratories (Mountain View, CA). The anti-PARP-1 antibody was purchased from Roche Applied Science (Indianapolis, IN). 3-PO was obtained from Chembridge Corporation (San Diego, CA) and generously provided by Dr. Brian Clem (University of Louisville, Louisville, KY).

### Cell Culture and Treatments

The immortalized bronchial epithelial cell line BEAS-2B and the NSCLC cell lines H1299, H661 and A549 were obtained from the American Type Culture Collection (Rockville, MD). The NSCLC cells were maintained in RPMI 1640 containing 10% FCS, 22 mM glucose, 10,000 units/ml penicillin, and 10,000 µg/ml streptomycin. The BEAS-2B cells were maintained in serum-free Bronchial Epithelial Cell Basal Medium (BMEM) in cultureware that has been precoated with collagen, fibronectin and albumin per the ATCC culturing protocol. All cell lines were maintained at 37°C in 5% CO_2_. Eighteen to twenty-four hours before treatment, 5000 cells per cm^2^ were seeded in 6-cm dishes to achieve 30% to 40% confluence at the time of treatment. This insured that the cells would not become confluent over a 72 h experimental period. Both the normal and cancer cells were treated with various concentrations of AA (25, 50, 100, 300, 500, 1000, 2000 µM) and 3-PO (1, 3, 10 and 30 µM) alone and in combination. Control and treated cultures were then processed according to the specific assay as described in the following sections.

### Trypan Blue Exclusion Assay

To assess cell viability 24 h post-treatment, floating cells were removed and retained and the plates were then trypsinized to release adherent cells. Both adherent and floating cells were combined and subjected to centrifugation (1,000 × g, 10 min at 4°C) and resuspended in 50 µl PBS containing trypan blue (0.4% solution). Viable cells were manually counted with a hemocytometer and a minimum of 200 cells were counted from each sample. The results presented are based on three independent experiments in which duplicate treatments were analyzed for each experiment.

### Detection of ROS Accumulation

Oxidative stress was assessed by measuring the intracellular levels of ROS generated after exposure of cells to AA or 3-PO or both. H1299 NSCLC and BEAS-2B lung epithelial cells were plated in 8 well chamber slides (20,000 and 10,000 cells per well, respectively), treated with either 300 µM AA, 10 µM 3-PO or a combination of AA and 3-PO, and were assayed at 15, 30, 60, 120, and 240 min post-treatment. After treatment, cells were washed with PBS and treated with 5 µM 5-(and-6)-chloromethyl-2′,7′-dichlorodihydrofluorescein diacetate, acetyl ester (CM-H_2_DCFDA, Life Technologies/Invitrogen, Grand Island, NY) at 37°C in the dark for 30 min to detect intracellular ROS. Cells were washed in PBS and observed using a fluorescent microscope using the FITC filter. The intensity of the fluorescence was measured from representative images using the AxioVision (Release 4.6.3) software. A total of 100–125 cells per treatment were measured in each experiment and the results presented represent two independent experiments (200–250 cells total per treatment).

### Assessment of H_2_O_2_-mediated Cytotoxicity

To determine whether H_2_O_2_ was required for the cytotoxic effects of combination treatments of AA and 3-PO, cells were treated with various doses of catalase (25, 50, 100, 200, 500, 1000, 2000 IU) or the catalase-specific inhibitor aminotriazole (30, 50,100 µM), in combination with AA (300 µM) and 3-PO (10 µM). Aminotriazole was added 1 h prior to the addition of AA and/or 3-PO and cell viability was assessed using the trypan blue exclusion assay. The results presented are based on three independent experiments in which triplicate treatments were analyzed for each experiment.

### Detection of DNA Fragmentation

DNA fragmentation was measured using both the comet [71] and TUNEL [72] assays. For the comet assay, cells were treated with 300 µM AA and 10 µM 3-PO, alone and in combination. At 24 h post-treatment, cells were collected by gently scraping the cells from the plate, followed by centrifugation at 1,000 × *g* for 5 min at 4°C. Cell pellets were resuspended in 1 ml of medium. Fully frosted microscopic slides were covered with 140 µl of 0.75% regular melting point agarose (40–42°C). After application of a coverslip, the slides were allowed to gel at 4°C for 10 min. Meanwhile, 20 µl of cell suspension was then added to 110 µl of 0.5% low melting point agarose (37°C). After carefully removing the coverslips a second layer of 110 µl of cell suspension mixture was pipetted onto the pre-coated slides and allowed to solidify at 4°C for 10 min. The coverslips were removed and a third layer of 110 µl of low melting point agarose was pipetted onto the slides and allowed to gel at 4°C for 10 min. The slides (without coverslips) were immersed in freshly prepared, cold lysing solution (2.5 M NaCl, 100 mM Na_2_EDTA, 10 mM Tris-HCl, pH 10, 1% sodium N-lauroyl sarcosinate, 1% Triton X-100 and 10% DMSO, DMSO added just before use) and refrigerated for 1 h. Slides were then placed in alkaline electrophoresis solution (300 mM NaOH and 1 mM EDTA, pH 13) for 20 min to allow unwinding of the DNA to occur. Electrophoresis was conducted for 25 min at 25 V (0.66 V/cm) and adjusted to 300 mA by raising or lowering the buffer level in the tank. Slides were then drained, placed on a tray and washed gently with three changes of 5 min each of neutralization buffer (0.4 M Tris-HCl, pH 7.5). DNA was precipitated and slides were dehydrated in absolute methanol for 10 min and were left at room temperature to dry. The whole procedure was carried out in dim light to minimize artifactual DNA fragmentation. All the slides were then stained with 50 µl of ethidium bromide (20 mg/ml) and viewed under a fluorescence microscope. Analysis was performed using a 40X objective. A total of 50 cells from each of three slides (150 cells total) were screened per treatment. Cells with DNA fragmentation have a comet-like appearance. The length of the DNA migration in the comet tail, which is an estimate of DNA fragmentation, was measured using CometScore Version 1.5 (TriTek Corp., Sumerduck, VA). The length of the comet tail is computed as the comet head diameter subtracted from the overall comet length. The results presented are based on three independent experiments in which triplicate treatments were analyzed for each experiment.

TUNEL assays were performed on H1299 cells plated in 8 well chamber slides (20,000 cells per well) treated with either 300 µM AA, 10 µM 3-PO or a combination of AA and 3-PO for 2, 4, 6, 12, 18 and 24 h. Cells were fixed in 4% methanol-free formaldehyde solution in PBS (25 min at 4°C), washed two times with PBS, permeabilized with 0.2% Triton X-100 (5 min), and rinsed twice with PBS. Detection of DNA fragmentation was performed by incubating cells for 60 min at 37°C in 50 µl of TUNEL incubation buffer (DeadEndTM Fluorometric TUNEL System; Promega, Madison, WI, USA). Analysis was performed using a fluorescent microscope. TUNEL-positive cells showed localized green fluorescence within the nucleus of apoptotic cells. A minimum of 100 cells were scored for each sample and the results are based on two independent experiments (≥ 200 cells total per treatment).

### Poly (ADP-ribose) Polymerase (PARP) Cleavage Assay

Protein extracts of treated or untreated cells grown in six-well plates were prepared by lysing cells in 100 µl/well cold RIPA buffer (Sigma-Aldrich, St. Louis, MO). Protein concentrations were determined using a bicinchoninic acid-based assay (BCA Protein Assay Reagent, Thermo Scientific, Rockford, IL). Protein extracts were fractionated by sodium dodecyl sulfate polyacrylamide gel electrophoresis (SDS-PAGE) using 10% PAGEr Gold Tris-Glycine PreCast gels (Lonza, Rockland, ME). After electrophoresis, proteins were transferred to Immobilon-P 0.45um PVDF membranes (Millipore, Billerica, MA) at 20V for 90 min at 4°C and the membrane was blocked with 5% (w/v) instant non-fat dry milk in Tris-Tween buffered saline (TTBS; 16.1 mM Tris-HCl/3.88 mM Tris-base/150 mM NaCl/0.5% Tween 20, pH 7.5). The blocked membrane was incubated with a 1∶3000 dilution of the anti-PARP-1 antibody in TTBS and incubated at 4°C over night. The filter was washed three times with TTBS followed by the incubation with a 1∶100,000 dilution of horse-radish peroxidase-conjugated goat anti-rabbit secondary antibody in 1% (w/v) instant non-fat dry milk in TTBS (Jackson ImmunoResearch, West Grove, PA). Duplicate immunoblots probed with an actin-specific antibody were used as loading controls. Bands were imaged using a Kodak Image Station 4000R Pro (Carestream, Rochester, NY, USA) and individual protein bands quantified using Carestream Molecular Imaging Software version 5.0. Data are expressed as intensity normalized to the appropriate actin control.

### Annexin V and Propidium Iodide Assay for Apoptosis

To evaluate apoptosis by assaying for annexin V binding in cells, cells were plated in 8 well chamber slides (20,000 cells per well), treated with either 300 µM AA, 10 µM 3-PO, or a combination of AA and 3-PO and were assayed 15 min, 30 min, 45 min, 60 min, 2 h, 3 h and 4 h post-treatment. Cells were rinsed with Apoalert binding buffer and resuspended in 200 µl of binding buffer. The chamber slides were then stained with 5 µl of DAPI to stain the DNA, 5 µl of annexin V (20 µg/ml in Tris-NaCl) to detect cells in early apoptotic stage and 10 µl of propidium iodide (50 µg/ml in Apolaert binding buffer) for detection of late apoptotic cells. Analyses were performed manually using a fluorescence microscope. Cells with bound annexin V show fluorescent green staining in the plasma membrane. Cells that have lost membrane integrity showed red staining throughout the cytoplasm and a halo of green staining on the cell surface (plasma membrane). A minimum of 200 cells were scored for each sample and the results are based on three independent experiments in which triplicate treatments were analyzed for each experiment.

### Caspase 3/7 Assay

To detect caspase activity in NSCLC cells, the CellEvent™ Caspase-3/7 Green Detection Reagent (Life Technologies/Invitrogen) was used. This assay utilizes a novel fluorogenic substrate for activated caspases 3 and 7. Apoptotic cells with activated caspases 3 and/or 7 will have bright green nuclei, while cells without activated caspases will have minimal fluorescence. For these experiments, cells were plated in 8 well chamber slides (20,000 cells per well), treated with either 300 µM AA, 10 µM 3-PO, or a combination of AA and 3-PO for 15 min, 30 min, 1 h and 2 h. The substrate was added and after a 30 min incubation at 37°C, the cells were observed under the fluorescence microscope. A total of 300–450 cells were analyzed for each experiment and the experiment was repeated three times, with triplicate treatments analyzed for each experiment.

### Statistical Analysis

Statistical analyses and determinations of the concentrations of test compounds required to inhibit cell growth by 50% (IC_50_) were done using GraphPad Prism version 5.04 (GraphPad Software, LaJolla, CA). Differences between the experimental groups were analyzed using 1-way or 2-way analysis of variance. All means were calculated from two or three experiments, and error bars represent SEM. All tests were two-sided, with significance being defined as 5% or less. Evaluation of compound interactions was performed using the median effect method [73] using CalcuSyn software (Biosoft, Cambridge, UK). For experiments where a single combination of AA and 3-PO was tested, synergism was assessed by calculating the Drewinko Index (DI) values [74] as previously described [75]. A DI  = 1 indicates additivity, a DI >1 indicates synergism, and a DI <1 indicates antagonism.

## Supporting Information

Figure S1 **ROS accumulation in control and treated BEAS-2B and H1299 cells.** BEAS-2B and H1299 cells were treated with either vehicle (control), AA (300 µM), 3-PO (10 µM) or a combination of AA and 3-PO for the indicated times. Cells were stained with 5 µM CM-H_2_DCFDA at 37°C in the dark for 30 min to detect intracellular ROS. Cells were imaged using a fluorescence microscope at 20X magnification using the FITC filter.
(TIF)
