# Supplementary figures and images for: Ascorbic Acid and a Cytostatic Inhibitor of Glycolysis Synergistically Induce Apoptosis in Non-Small Cell Lung Cancer Cells
Source: PLoS One. 2013 Jun 11;8(6):e67081. doi: 10.1371/journal.pone.0067081 (PMC3679078; doi:10.1371/journal.pone.0067081)

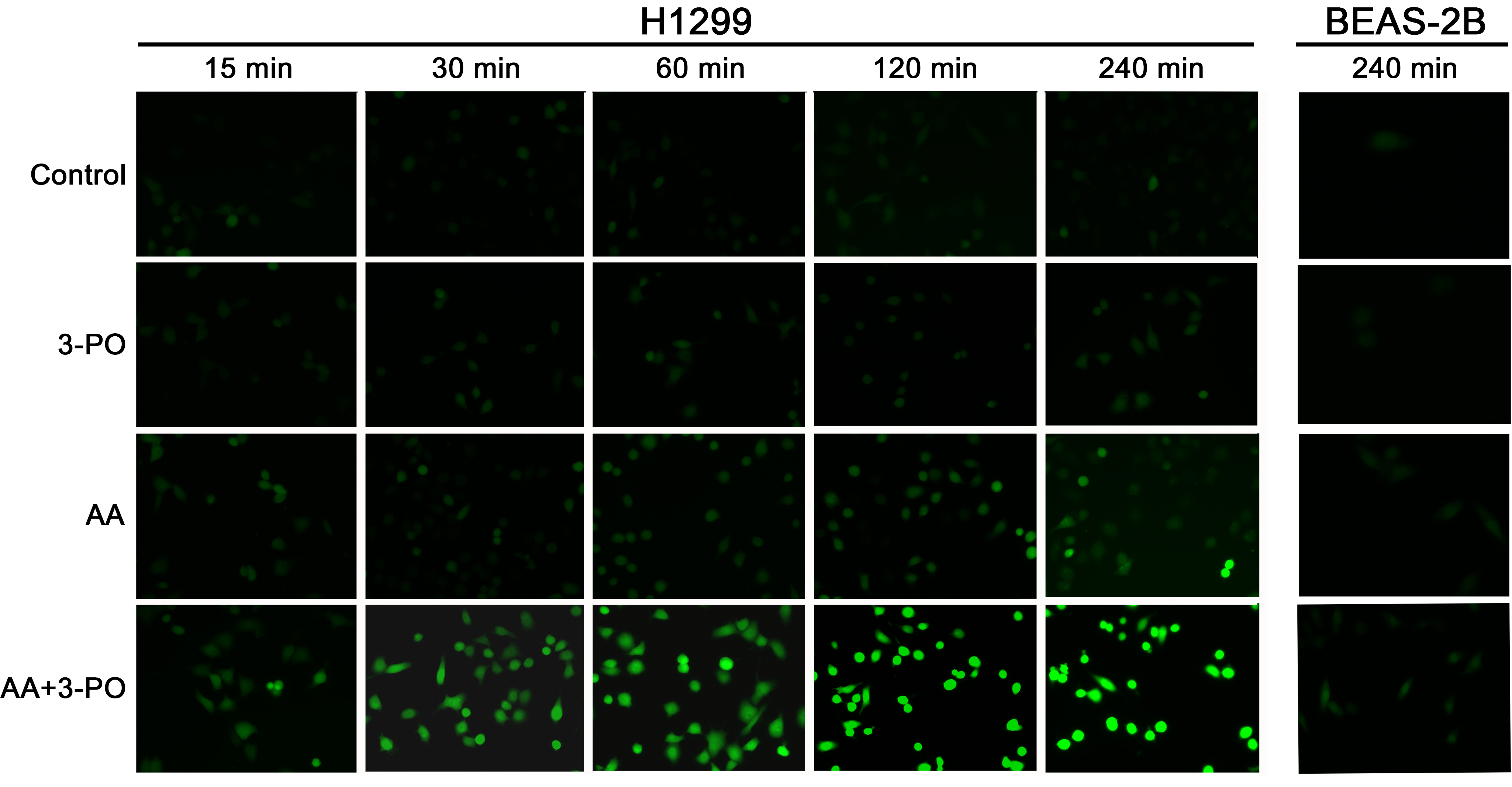

Supplement: Figure S1 — ROS accumulation in control and treated BEAS-2B and H1299 cells. BEAS-2B and H1299 cells were treated with either vehicle (control), AA (300 µM), 3-PO (10 µM) or a combination of AA and 3-PO for the indicated times. Cells were stained with 5 µM CM-H2DCFDA at 37°C in the dark for 30 min to detect intracellular ROS. Cells were imaged using a fluorescence microscope at 20X magnification using the FITC filter. (TIF) [file pone.0067081.s001.tif]
